# The Role of the Axillary Arch Variant in Neurovascular Syndrome of Brachial Plexus Compression

**DOI:** 10.7759/cureus.2875

**Published:** 2018-06-25

**Authors:** Rabjot Rai, Joe Iwanaga, Marios Loukas, Rod J Oskouian, R. Shane Tubbs

**Affiliations:** 1 Anatomy, St. George's University School of Medicine, St. George's, GRD; 2 Medical Education and Simulation, Seattle Science Foundation, Seattle, USA; 3 Anatomy, St. George's University, St. George's, GRD; 4 Neurosurgery, Swedish Neuroscience Institute, Seattle, USA; 5 Neurosurgery, Seattle Science Foundation, Seattle, USA

**Keywords:** axillary arch, langer’s arch, brachial plexus compression, neurovascular, median nerve

## Abstract

Axillary arch muscles are often found. In their course through this area, they might interfere with regional neurovascular structures. This case report will examine the presence of the axillary arch muscle and its implication in brachial plexus compression. During routine dissection of the left axilla and upper limb, a variant muscle (axillary arch muscle) was identified arising from the distal tendon of the latissimus dorsi and extending laterally to insert onto the deep surface of the tendon of insertion of the deltoid muscle. In adduction of the upper limb, the muscle was lax without compression of any underlying neurovascular structures. However, in abduction, the aberrant band of muscles compressed the proximal branches of the brachial plexus. Clinicians should be aware of this anatomical variant and its clinical significance in neurovascular compression including brachial plexus compression, thoracic outlet syndrome, and hyperabduction syndrome. This literature will review the anatomy of the axillary arch and its clinical correlate regarding signs, symptoms, diagnosis, and treatment in brachial plexus compression.

## Introduction

The most common anatomical variant found in axilla is the axillary arch muscle, known by various terms including: Langer’s arch, Langer’s muscle, achselbogen, axillopectoral muscle, arcus axillans, and pectorodorsalis muscle [1-3]. This variant is described as a thin flat musculotendinous band originating from the anterior portion of the latissimus dorsi and traveling anteriorly to insert into various regions of the upper extremity [4]. This anatomical variant is reportedly present in 7% of the general population; however, other studies report varying ranges of 0.25% to 37.5% [1, 4]. A higher prevalence of 43.8% is reported in Chinese populations, and the muscle is noted to be more common in females than males [2, 5].

There are multiple variations of the axillary arch variant identified in the literature in terms of its size, structure, tissue composition, and course of insertion. The axillary arch varies in length from 7 to 10 cm and 5 to 15 mm in width [6, 7]. It commonly occurs bilaterally, but can be observed unilaterally. Normally, the axillary arch presents as a bidirectional band with one origin and one insertion point, however, double and rarely multiple bands toward insertion points may occur [1, 2]. There are two types of tissues involved in the formation of the axillary arch depending on its major contributor: 1) muscular, where the majority is formed from the pectoralis major muscle; 2) tendinous, where the latissimus dorsi muscle is its major contributor [8]. The axillary arch is classified based on its complete or incomplete forms. In the more common complete form, the arch arises from the latissimus dorsi and extends to the trilaminar tendon on the pectoralis major located on the humerus. On the other hand, the incomplete form arises from the latissimus dorsi with insertion onto variable regions including the axillary fascia, inferior margin of pectoralis minor, coracobrachialis muscle, coracobrachial fascia, long and short heads of the biceps brachii, teres major, coracoid process, and first rib [2, 8]. Variations in the shape of the axillary arch have also been detailed in the works of Dharap—the axillary arch was described as triangular muscle—and by Serpell and Baum—the muscle was defined as fusiform in shape [9].

Although the embryological origins of the axillary arch muscles are uncertain, theory suggests an association with the development of the panniculus carnosus, a remnant of a “skin-associated musculature” found between the subcutaneous fat and superficial fascia. The panniculus carnosus is highly developed in lower mammals, such as rodents and cats. In humans, evolution preferred wider upper limb mobility rendering the panniculus to a vestigial structure. The platysma and dartos muscles are remnants of the panniculus and are found sporadically in humans [1, 2, 10]. The axillary arch provides no functional significance and this absence of importance may clarify the numerous variations identified [2].

Regardless of the lack of functionality, the axillary arch muscle is implicated in clinical syndromes and its importance is stressed during surgical application in the axilla. Brachial plexus impingement, thoracic outlet syndrome, hyperabduction syndrome, shoulder instability, and venous obstructive compression can occur secondary to the axillary arch muscle [1, 2, 4, 5]. While surgically, the axillary arch may obscure proper visualization of the lymph nodes during lymphadenectomy of breast carcinoma rendering incomplete clearance [1]. Currently, reports in the literature regarding neurovascular impingement by the axillary arch muscle are limited and sporadic. This case report will also review the role of axillary arch muscle in neurovascular compression of the brachial plexus. The anatomy regarding the innervation of the axillary arch, and the clinical significance concerning signs, symptoms, diagnosis and treatment of brachial plexus compression by such muscles will be discussed.

## Case presentation

**Figure 1:**
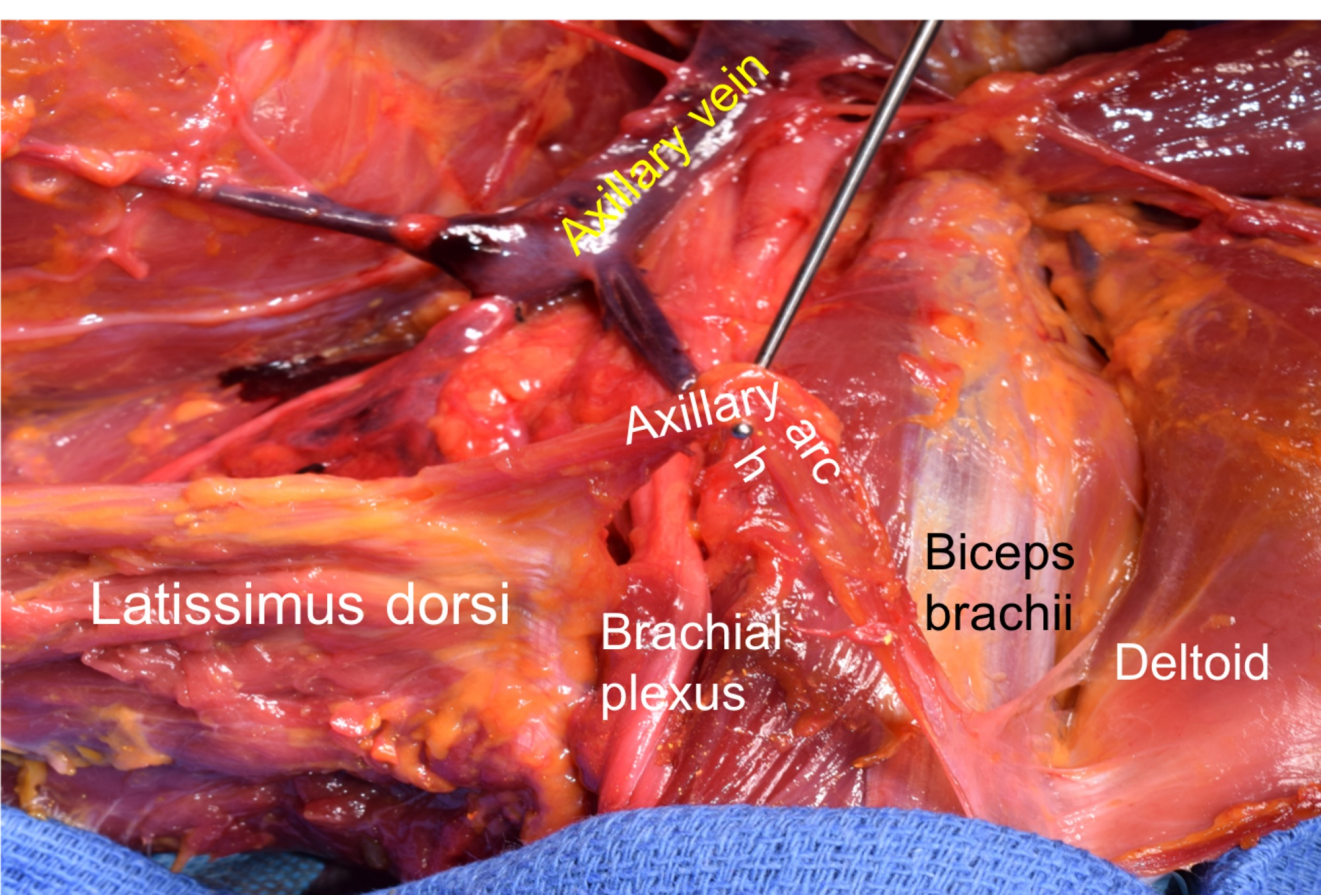
Case reported herein illustrating the left axilla and proximal upper limb. Note the lax axillary arch muscle being lifted with the dissector. The attachments of this aberrant muscle are seen at the latissimus dorsi and deltoid muscles.

**Figure 2:**
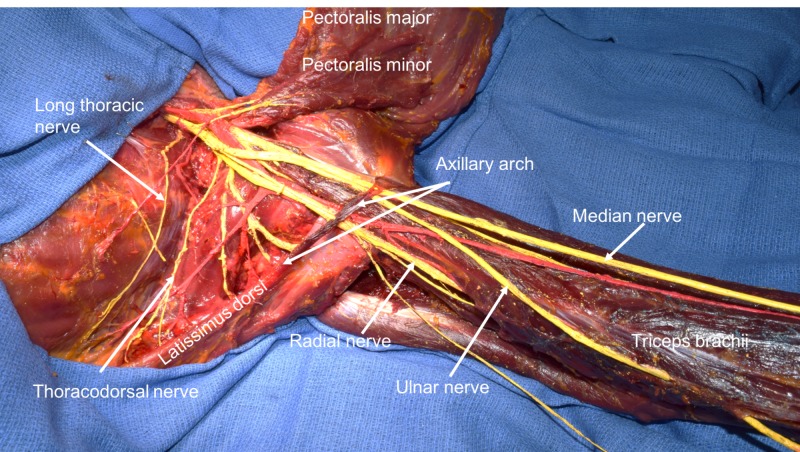
Abduction of the upper limb and note the compression of underlying nerves by the axillary arch muscle.

During routine dissection of the left axilla and upper limb, a variant muscle (axillary arch muscle) was identified arising from the distal tendon of the latissimus dorsi and extending laterally to insert onto the deep surface of the tendon of insertion of the deltoid muscle (Figure 1). The fresh frozen specimen was, at death, an 83-year-old female. In adduction of the upper limb, the muscle was lax without compression of any underlying neurovascular structures. However, in abduction, the aberrant band of muscles compressed the proximal branches of the brachial plexus. Specifically, the most compressed of these underlying nerves were the median, ulnar and radial nerves (Figure 2). The compression was significant enough to efface each of these nerves by 50%, especially the median and ulnar nerves. No atrophy of distal muscles innervated by these nerves was appreciated. No axillary arch muscle was found on the contralateral side of the cadaver. No other musculoskeletal or neurovascular variants were seen during further dissection of these areas. No specific nerve innervation to the axillary arch muscle was identified.

## Discussion

The flat musculotendinous slip of the axillary arch originating from the latissimus dorsi often travels across the neurovascular structures of this region. There are various reports regarding the innervation of the axillary arch. Certain literature suggests innervation of the axillary arch is more common from the medial pectoral nerve, as its embryological origin is related to the pectoral muscle mass. However, the thoracodorsal nerve is also noted to innervate the axillary arch due to its close association to the latissimus dorsi. Other various innervations reported in previous studies include the intercostobrachial nerve, and medial cutaneous nerve of the arm [6, 9-11].

A study conducted by Guy et al. examined the relationship between the axillary arch and neurovascular bundle. All cases demonstrated the axillary arch arising inferior to the neurovascular bundle and crossing anteriorly over the bundle before inserting onto sites in the upper extremity. The study evaluated the distance between the axillary arch muscle and the neurovascular bundle (brachial artery, vein or nerve plexus). The results found that 70% of patients were within 5 mm, while 25% were within 1 mm, and some patients displayed no visible gap between the two structures [4]. The proximity to the neurovascular bundle highlights the possibility of brachial plexus compression via axillary arch muscles.

Neurovascular compression by the axillary arch muscle is noted at the cervico-axillary region, which houses the passageway of the brachial plexus, long thoracic nerve, and axillary blood vessels [5]. Patients with brachial plexus compression via axillary arch muscles report symptoms similar to thoracic outlet syndrome. Symptoms range from painful paresthesias of the arm and upper extremity swelling to weakness. Exacerbation of pain is noted with abduction, external rotation, and elevation of the shoulder [12-14]. Due to the anterior location of the axillary arch, the median nerve and its medial and lateral cords are the most likely brachial plexus regions to be impinged. Patients would report numbness and pain in the anterior forearm or lateral part of the hand [4]. During abduction and external rotation, the musculotendinous band is taut and compresses underlying structures such as the median, ulnar, and musculocutaneous nerves [12]. The entrapment of vessels and nerves by the axillary arch may present with various symptoms. The compression of vasculature may resemble hyperabduction syndrome with venous distension, edema, and slight neurological and arterial involvement [5].

The presence of an axillary arch on physical examination of the axilla will be seen as loss of concavity, palpable firm mass, or noticeable fullness of the axilla [1, 5]. Patients with concurrent intermittent axillary vein obstruction will present with signs of swelling, discoloration, and lymphedema [4]. Axillary masses may be mistaken for enlarged lymph nodes or a soft tissue tumor, thus the diagnosis should be confirmed with imaging.

Multiple imaging modalities have been used to diagnose the presence of the axillary arch. In the current literature, the use of magnetic resonance imaging (MRI), magnetic resonance axillography, and phlebography due to axillary vein compression has been utilized for the diagnosis [5]. Also, Clarys et al. reported the use of echography in obese patients for diagnosis, providing an inexpensive approach [15]. Incidental findings of the axillary arch are infrequent and few, with reports of seeing the muscle during venography, mammography, computed tomography (CT), and MRI. The few incidental reports are due to the positioning of the patient. Normally, the patients’ arms are to their side, allowing imaging through the brachial plexus, thus to visualize the axillary arch the patient’s arm needs to be in an abducted position [13]. Once identified, the superficial nature of the arch is easily accessed without difficulty and excised for the complete resolution of symptoms [5, 13].

There have been a limited number of cases where the axillary arch muscle compressed underlying neurovascular structures. One reason is that the arch is rarely identified or sought by clinicians. The importance of acknowledging axillary arch muscles as a differential diagnosis to brachial plexus impingement was also emphasized by Guy et al. who noted greater documented but unknown reasons of upper extremity numbness and radiating pain in patients with an axillary arch than those without [4]. The upper extremity neurological findings in these patients were attributed to the proximity of the axillary arch to the brachial plexus. The resolution of symptoms following excision of the musculotendinous band further reinforced the clinical importance of the axillary arch. The added cognizance of the existence of the axillary arch will provide clinicians with a wider differential set for brachial plexus compression.

## Conclusions

Although the axillary arch is a vestigial structure, its clinical importance remains noteworthy. The axillary arch is reported in various neurovascular compression syndromes, including thoracic outlet syndrome, hyperabduction, and brachial plexus impingement. Awareness of the axillary arch variant will better inform clinicians to consider it as a differential diagnosis in patients with unidentified upper extremity neurovascular compression symptoms. Diagnosis should be confirmed with imaging and excision of the variant is usually curative.
